# Anesthesia upstream of the alcoholic lesion point alleviates the pain of alcohol neurolysis for intercostal neuralgia: a prospective randomized clinical trial

**DOI:** 10.6061/clinics/2020/e1296

**Published:** 2020-01-14

**Authors:** Jiyu Kang, Yang Liu, Li Niu, Mengli Wang, Chao Meng, Huacheng Zhou

**Affiliations:** I Department of Anesthesiology, the Fourth Affiliated Hospital of Harbin Medical University, Harbin, China; II Department of Anesthesiology, the Huludao Central Hospital, Huludao, China; III Department of Anesthesiology, the 211 Hospital of Chinese PLA, Harbin, China; IV Department of Pain Management, the Affiliated Hospital of Qingdao University, Qingdao, China

**Keywords:** Intercostal Neuralgia, Alcohol, Neurolysis

## Abstract

**OBJECTIVES:**

Alcohol for intercostal neuralgia may induce severe injection pain. Although nerve block provided partial pain relief, alcohol might be diluted, and the curative effect decreased when the local anesthetic and alcohol were given at the same point. Therefore, we observed the modified method for intercostal neuralgia, a Two-point method, in which the local anesthetic and alcohol were given at different sites.

**METHOD:**

Thirty patients diagnosed with intercostal neuralgia were divided into 2 groups: Single-point group and Two-point group. In the Single-point group, alcohol and local anesthetic were injected at the same point, named the “lesion point”, which was the lower edge of ribs and 5 cm away from the midline of the spinous process. In the Two-point group, alcohol was injected at the lesion point, whereas the local anesthetic was administered at the “anesthesia point”, which was 3 cm away from the midline of spinous process.

**RESULTS:**

After alcohol injection, visual analog scale (VAS) in the Two-point group was lower than the Single-point group, and the satisfaction ratio of patients in the Two-point group was higher (*p*<0.05). The degree of numbness in the Two-point group was greater than the Single-point group at 1 month and 3 months after operation (*p*<0.05). However, the long-term effects did not differ.

**CONCLUSIONS:**

Local anesthetic was given upstream of the point where alcohol was administered, was a feasible and safe method to relieve pain during the operation, and improved the satisfaction of the patients and curative effect.

## INTRODUCTION

Intercostal neuralgia refers to thoracic and abdominal pain with a zonal distribution caused by intercostal nerve impairment, which can be induced by different stimuli, such as trauma, tumor, neuroma, or herpes zoster (1,2). Intractable intercostal neuralgia often manifests as a sharp or burning pain accompanied by exacerbation when coughing and sneezing, and pain to the touch (2), which significantly reduces the quality of life. Many methods have been adopted to treat intractable intercostal neuralgia, including drugs (3), nerve block (4-6), and pulsed radiofrequency (7). However, the conventional operations were not effective enough, and they often needed repeating and offered short-term therapeutic effects.

Neurolysis, such as radiofrequency ablation (8), cryoablation (9), and chemical lesion (10), could bring long-term effects. However, radiofrequency ablation and cryoablation require specialized equipment, longer operation times, and higher expenses. Chemical lesions, such as with alcohol and phenol (11,12), have the advantages of convenient operation and good effectiveness, and no difference in pain outcomes was found between alcohol- and phenol-based neurolytic techniques (13). In contrast, the neurolytic agents stimulated tissues too strongly and induced severe pain when injected, which affected the cooperation and satisfaction of patients during the operation (14,15). Traditionally, local anesthetic, such as lidocaine or ropivacaine, was given to relieve the pain before applying alcohol at the same injection site (Single-point method), but this procedure might dilute alcohol and weaken the long-term curative effect (14). Therefore, we applied intercostal nerve block at a location upstream of the nerve lesion site, followed by alcohol injection at the lesion point. This method was named the “Two-point method”. This study will compare the curative effect and the satisfaction of patients between the Single-point method and the Two-point method.

## MATERIAL AND METHODS

### Patients

This study was approved by the Ethics Committee of the Fourth Affiliated Hospital of Harbin Medical University, and all patients provided their informed consent.

A total of 33 patients (19 male and 14 female) who suffered from intractable intercostal neuralgia and were hospitalized in the Fourth Affiliated Hospital of Harbin Medical University between Dec 2014 and Dec 2015 were enrolled in this study. Inclusion criteria: (1) age from 50 to 80 years old; (2) American Society of Anesthesiologists (ASA) grade I to III; (3) successful pain relief after a diagnostic block with local anesthetic. Exclusion criteria: (1) serious medical diseases such as incompetence of the heart, lung or kidney; (2) communication barriers and uncooperative cases; (3) local or systemic infection; (4) allergy to anesthetics; and (4) coagulation abnormality. The patients were divided into 2 groups: the Single-point group (n=16) and the Two-point group, by a random number method (n=17). The number of neurolysis of intercostal nerve was determined by the patient's pain range.

### Single-point group procedure

**Figure 1:**
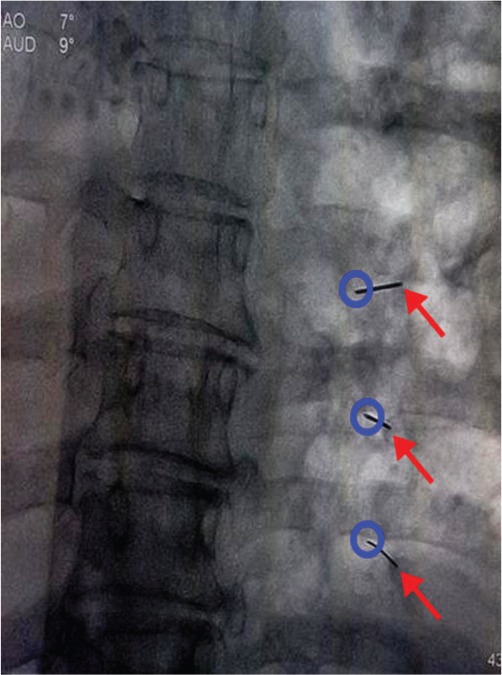
The setup of the Single-point group. The red arrows refer to the puncture needles. The blue circles are shown as the lesion point.

The operation was performed under X-ray guidance. Patients were placed in a prone position. First, the targeted intercostal nerve-corresponding rib was located. Then, a point, named the “lesion point”, at the lower edge of the ribs and 5 cm away from the midline of the spinous process was marked on the skin (Figure 1). After aseptic technique and local infiltration anesthesia with 1% lidocaine and 0.5% ropivacaine, a needle (22 G) was punctured vertically at the lesion point until it reached the rib bone, then the needle tip was slid the lower edge of the rib and advanced about 2-3 mm. Then, 1 ml of 1% lidocaine and 0.5% ropivacaine without vasoconstrictor was administered, and 1 ml dehydrated alcohol (Tianjin TIANLI Chemical Reagents Ltd., Tianjin, China) was injected 3 min later. Finally, the needle was pulled out and a sterile dressing was pasted. The same procedure was performed on another intercostal nerve if necessary.

### Two-point group procedure

**Figure 2:**
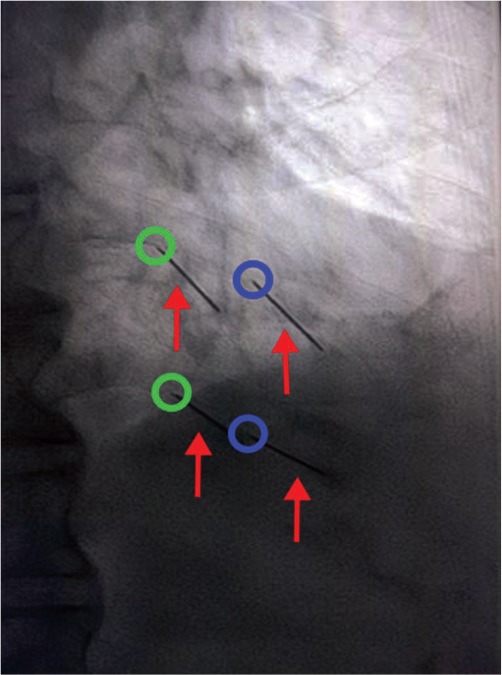
The setup of the Two-point group. The red arrows refer to the puncture needles. The blue circles are shown as the lesion point, and the green circles are shown as the anesthesia point.

Patients in the Two-point group were placed in the same position as those in the Single-point group. In addition to the lesion point, a point that at the lower edge of the ribs and 3 cm away from the midline of the spinous process was marked on the skin, named the “anesthesia point” (Figure 2). After aseptic technique and local infiltration anesthesia with 1% lidocaine and 0.5% ropivacaine, a needle (22 G) was punctured at the anesthesia point and another at the lesion point. At the anesthesia point, 1 ml of 1% lidocaine and 0.5% ropivacaine was injected. After 3 min, the 1 ml dehydrated alcohol was injected at the lesion point. The rest of the operation was the same as the Single-point method.

### Measurements

The demographic data was recorded. The 10-point VAS (0 for no pain and 10 for unbearable pain), the degrees of numbness (None; slight; moderate; and severe) (16), the mean arterial pressure (MAP), and heart rate (HR) were recorded before the operation (baseline) and at 1 min (immediate assessment after alcohol given), 2 h, 24 h, 1-month, and 3-month after the operation. At 1 min after alcohol injection, the satisfaction of patients was assessed. According to the degree of pain postoperatively, patients could take tramadol tablets (100 mg once, every 4-6 h; maximum dose of 400 mg). Complications, including paresthesia, aggravation of the pain, pleural reaction, pneumothorax, local hematoma, infection, and local anesthetic toxicity, were also recorded.

### Statistical analysis

With the respect to the VAS at 1 min after alcohol injection (6.25 in the Single-point group, 2.75 in the Two-point group), a sample size of 10 patients per group was required to provide 80% power to detect differences at an α level of 0.05 to indicate significance.

The results are presented as the means±standard deviation (SD), number, and percentage. A normality test was performed before the statistical analysis with t test. The comparisons were performed using the chi-square test or Fisher’s exact test for count data (gender, ASA grade, and treatment level), and the independent-sample t-test for measurement data (age, BMI, MAP, and HR). The change in VAS was analyzed by Two-way analysis of variance. The degree of numbness and the satisfaction of patients were analyzed by repeated-measures analysis of variance. *p*<0.05 was considered statistically significant. SPSS 20.0 was used to perform statistical analysis.

## RESULTS

**Figure 3:**
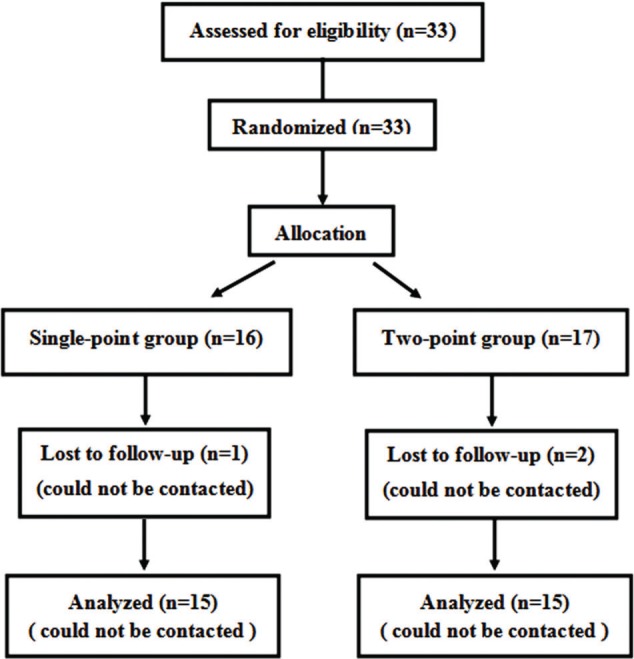
Flow diagram of the randomized controlled trial.

As 1 patient in the Single-point group and 2 patients in the Two-point group were lost to follow-up, 15 patients in the Single-point group and 15 patients in the Two-point group were eventually included in this study (Figure 3).

The demographic data in the two groups had no significant difference. Three intercostal nerves were destroyed in 9 patients and 2 were destroyed in 6 patients in the Single-point group, and 3 intercostal nerves were destroyed in 10 patients and 2 were destroyed in 5 patients in the Two-point group, which had no significant difference (Table 1). All the patients were operated on successfully.

The patients in the Single-point group had severe pain at the moment of alcohol injection, but the pain in the Two-point group was significantly relieved at 1 min (Single-point group VAS: 7.73±1.75; Two-point group VAS: 1.93±1.49; *p*<0.05). In each group, the VAS score at 2 h, 24 h, 1-month, and 3-month after the operation decreased significantly compared to that at baseline (*p*<0.05). However, the VAS at 2 h, 24 h, 1-month, and 3-month after operation in the Two-point group did not significantly differ from the Single-point group (Table 2).

MAP and HR exhibited the same trends as the VAS. The MAP and HR at 1 min after operation in the Two-point group (98.9±7.2 mm Hg and 79.1±8.5 rates/min) were lower than those in the Single-point group (110.2±6.8 mm Hg and 92.5±8.9 rates/min), respectively (*p*<0.05 for both). The MAP and HR at 2 h, 24 h, 1-month, and 3-month after operation had no statistical difference between the two groups (data were not shown).

The degree of numbness was aggravated in both groups with time, and the degree of numbness at 1-month and 3-month after the operation in the Two-point group was greater than the Single-point group (*p*<0.05, Table 3). The satisfaction ratio of patients at 1 min and 2 h after the operation in the Two-point group was higher than in the Single-point group (*p*<0.05), while the satisfaction ratio at 24 h, 1-month and 3-month after the operation in both groups did not significantly differ (Table 4).

Five and 3 patients took drugs postoperatively in the Single-point group and the Two-point group, respectively, which did not significantly differ. Additionally, no serious complications were observed in any patients. However, 2 patients in the Single-point group exhibited aggravation of the pain, and 2 patients in the Two-point group exhibited paresthesia. There was no significant difference between the two groups (Table 5).

## DISCUSSION

In this prospective randomized clinical trial, intercostal neurolysis was successfully implemented in all patients. The results showed that the Two-point method decreased the VAS score, MAP, and HR during the operation and improved the satisfaction of patients compared with the Single-point method.

Many kinds of neurolytic agents are applied in the clinic, with alcohol being the most widely used (17). Dehydrated alcohol is a colorless transparent liquid, and the concentration is greater than 99.5% when used (17). The alcohol can contact nerves directly, destroying the nerve structure and blocking nerve conduction, providing the patients with pain relief (18). Neurolysis with alcohol is used to reduce pain induced by different causes. In this study, intercostal neurolysis with alcohol decreased the pain of patients as indicated by the reduced VAS score, which meant that alcohol was effective in the treatment of intercostal neuralgia. Hung et al. (14) reported that neurolytic transversus abdominal plane block with alcohol offered long-term control of malignant abdominal wall pain. Fujita (19) found that splanchnic nerve neurolysis with alcohol decreased upper abdominal cancer pain. Chen et al. (20) indicated that alcohol neurolysis of lateral femoral cutaneous nerve provided sustained pain relief for recurrent meralgia paresthetica.

However, alcohol can induce severe pain after injection (15). The effect of alcohol is felt rapidly and can induce severe temporary pain due to local irritation. Transient severe pain could cause severe complications, such as diseases of the circulatory system and nervous system. Thus, elimination of the pain induced by alcohol during the operation is necessary.

Nerve block before alcohol injection at the same site can partially relieve the pain during the operation (10,21), as corroborated by this study. However, the Single-point method still induced severe pain during the operation, as shown by the increased VAS score, MAP, and HR. This pain does not mean inadequate anaesthesia. The effect might have at least two causes. First, the concentrations of alcohol and local anesthetic were both diluted when admitted at the same site, which decreased the analgesic effect of local anesthetic. Second, when the alcohol was given at the same point as the anesthesia, the diffusion range of alcohol may be widened by the anesthetic injected previously, and the stimulation of the surrounding tissue may be more serious. Thus, the Two-point method was proposed.

In the Two-point method, the lesion site was covered by the full effect of the nerve block, so the pain induced by alcohol during the operation was reduced. And the concentration of alcohol was not affected by the previous local anesthetic injection, so the effect of alcohol neurolysis was maximized. These factors combined to reduce the pain after the alcohol injection and improved the patients’ satisfaction. Moreover, the degree of numbness at 1-month and 3-month after operation were more obvious, and the curative effect was more stable and precise.

The alcohol concentration is very important in the curative effect. The degree of nerve injury can be increased by increasing the concentration of alcohol (22,23). Wang et al. (24) found that the effect of 99.9% alcohol on the rat sciatic nerve was more obvious than 50% or 75% alcohol, and the nerve recovery lasted for more than 12 weeks. Hung et al. (14) reported that a higher concentration of alcohol provided longer pain relief than a lower concentration for neurolytic transversus abdominal plane block with alcohol. The Two-point method avoided the risk of alcohol dilution, giving it the advantages of a stronger effect and longer action time.

A few limitations of this study were worth noting. First, the sample size was small, and the results needed further support by a larger sample. Second, only alcohol was used in this study; the effects might be influenced by the individual differences in drug sensitivity. Thirdly, patients under the one-point group referred high VAS (>7) at 1 min, which lasted for about 30s and can be tolerated by patients. This may cause a bias. If adequate anesthetic effects were provided during the one-point group, the alcohol concentration would be diluted and the long-term effect reduced, which proved the practicality and significance of the two-point method more. Fourthly, the distribution of drug was not clear due to that the contrast agent was not used. Finally, the follow-up time was only 3 months, and a longer time might reveal more benefits or drawbacks.

## CONCLUSION

Nerve block upstream of the nerve lesion point decreased the acute pain induced by alcohol during the operation and improved the patients’ satisfaction and the long-term curative effect. The Two-point method could be popularized in clinical practice.

## AUTHOR CONTRIBUTIONS

Meng C, Zhou H and Kang J conceived and designed the study, and were responsible for the manuscript drafting, and critical review for important intellectual content. Meng C, Zhou H, Liu Y, Niu L and Wang M were responsible for the acquisition of data. Kang J and Liu Y provided the analysis and interpretation of data. Meng C, Zhou H, Kang J, Liu Y, Niu L and Wang M were responsible for the approval of the final version of the manuscript to be submitted.

## Figures and Tables

**Table 1:** General data (mean±SD, n=15).

	Single-point group	Two-point group
Female/Male (number)	6/9	8/7
Age (y)	61.3±8.7	62.3±11.0
BMI (kg/m^2^)	24.5±2.5	24.0±2.5
ASA grade		
I	4	5
II	8	9
III	3	1
MAP (mm Hg)	99.6±6.4	99.9±7.0
HR (rates/min)	76.9±8.7	77.9±9.3
Treatment levels (number)		
IN 3-5	3	5
IN 4-6	4	2
IN 5-7	2	3
IN 5-6	3	3
IN 6-7	3	2

The general data of patients in the two groups had no significant difference.

BMI: body mass index; ASA: American Society of Anesthesiologists; MAP: mean arterial pressure; HR: heart rate; IN: intercostal nerve.

**Table 2:** The VAS at different time points (mean±SD, n=15).

	Baseline	1 min	2 h	24 h	1-month	3-month
Single-point group	7.27±0.96	7.73±1.75	3.00±0.65#	2.93±0.70#	2.73±1.10#	2.67±0.72#
Two-point group	7.47±0.99	1.93±1.49* #	2.67±0.72#	2.47±0.83#	2.40±0.74#	2.27±0.96#

Baseline: before the operation; 1 min: 1 min after alcohol given. 2 h, 24 h, 1-month, and 3-month represented the following time points: 2 h, 24 h, 1-month, and 3-month after the operation. VAS: visual analog score.

* *p*<0.05 *vs*. Single-point group;

# *p*<0.05 *vs*. baseline.

**Table 3:** The degrees of numbness (number (percentage)).

		Baseline	1 min	2 h	24 h	1-month	3-month
Single-point group	Severe	0 (0.0%)	0 (0.0%)	0 (0.0%)	2 (13.3%)	1 (6.7%)	3 (20.0%)
	Moderate	0 (0.0%)	3 (20.0%)	5 (33.3%)	5 (33.3%)	6 (40.0%)	8 (53.3%)
	Slight	0 (0.0%)	6 (40.0%)	8 (53.4%)	7 (46.7%)	7 (46.6%)	4 (26.7%)
	None	15 (100%)	6 (40.0%)	2 (13.3%)	1 (6.7%)	1 (6.7%)	0 (0.0%)
Two-point group	Severe	0 (0.0%)	0 (0.0%)	1 (6.7%)	4 (26.6%)	7 (46.7%)	9 (60.0%)
	Moderate	0 (0.0%)	4 (26.6%)	6 (40.0%)	7 (46.8%)	6 (40.0%)	6 (40.0%)
	Slight	0 (0.0%)	6 (40.0%)	6 (40.0%)	4 (26.6%)	2 (13.3%)	0 (0.0%)
	None	15 (100%)	5 (33.4%)	2 (13.3%)	0 (0.0%)	0 (0.0%)	0 (0.0%)
*p*		1.000	1.000	0.877	0.445	0.035*	0.023*

The degrees of numbness included 4 degrees: severe, moderate, slight, and none. Severe = 7 to 10 scores; moderate = 3 to 6 scores; slight = 1 to 2 scores; none = 0 score. Baseline: before the operation; 1 min: 1 min after alcohol given. 2 h, 24 h, 1-month, and 3-month represented the following time points: 2 h, 24 h, 1-month, and 3-month after the operation.

* *p*<0.05 *vs*. Single-point group.

**Table 4:** The satisfaction of patients (number (percentage)).

		Baseline	1 min	2 h	24 h	1-month	3-month
Single-point group	Extreme	N	2 (13.3%)	3 (20.0%)	7 (46.7%)	10 (66.7%)	12 (80.0%)
	Moderate	N	11 (73.4%)	10 (66.7%)	7 (46.7%)	5 (33.3%)	3 (20.0%)
	No	N	2 (13.3%)	2 (13.3%)	1 (0.07%)	0 (0.0%)	0 (0.0%)
Two-point group	Extreme	N	9 (60.0%)	10 (66.7%)	12 (80.0%)	14 (93.3%)	14 (93.3%)
	Moderate	N	6 (40.0%)	5 (33.3%)	3 (20.0%)	1 (0.07%)	1 (0.07%)
	No	N	0 (0.0%)	0 (0.0%)	0 (0.0%)	0 (0.0%)	0 (0.0%)
*p*		N	0.021*	0.025*	0.128	0.169	0.598

N: the satisfaction was not measured. Baseline: before the operation; 1 min: 1 min after alcohol given. 2 h, 24 h, 1-month, and 3-month represented the following time points: 2 h, 24 h, 1-month, and 3-month after the operation.

* *p*<0.05 *vs*. Single-point group.

**Table 5:** Results of drug usage and complications (number (percentage)).

	Single-point group	Two-point group	*p*
Drug usage			0.682
Yes	5 (33.3%)	3 (20.0%)	
No	10 (66.7%)	12 (80.0%)	
Complications			1.000
Yes	2 (13.3%)	2 (13.3%)	
No	13 (86.7%)	13 (86.7%)	

There were 5 cases taking drugs postoperatively in the Single-point group and 3 cases in the Two-point group, which had no significant difference. There were 2 cases of aggravation of the pain in the Single-point group, and 2 cases of paresthesia in the Two-point group. *p<0.05 *vs*. Single-point group.

## References

[1] Bajwa ZH, Sami N, Warfield CA, Wootton J (1999). Topiramate relieves refractory intercostal neuralgia. Neurology.

[2] Olmarker K, Rydevik B (2001). Selective inhibition of tumor necrosis factor-alpha prevents nucleus pulposus-induced thrombus formation, intraneural edema, and reduction of nerve conduction velocity: possible implications forfuture pharmacologic treatment strategies of sciatica. Spine.

[3] Karmakar MK, Ho AM (2004). Postthoracotomy pain syndrome. Thorac Surg Clin.

[4] Britt T, Sturm R, Ricardi R, Labond V (2015). Comparative evaluation of continuous intercostal nerve block or epidural analgesia on the rate of respiratory complications, intensive care unit, and hospital stay following traumatic rib fractures: a retrospective review. Local Reg Anesth.

[5] Doi K, Nikai T, Sakura S, Saito Y (2002). Intercostal nerve block with 5% tetracaine for chronic pain syndromes. J Clin Anesth.

[6] Xiao P, Zhu X, Wu X (2014). Curative effect research on curing intercostal neuralgia through paravertebral nerve block combined with pregabalin. Pak J Pharm Sci.

[7] Cohen SP, Sireci A, Wu CL, Larkin TM, Williams KA, Hurley RW (2006). Pulsed radiofrequency of the dorsal root ganglia is superior to pharmacotherapy or pulsed radiofrequency of the intercostal nerves in the treatment of chronic postsurgical thoracic pain. Pain Physician.

[8] Engel AJ (2012). Utility of intercostal nerve conventional thermal radiofrequency ablations in the injured worker after blunt trauma. Pain Physician.

[9] Byas-Smith MG, Gulati A (2006). Ultrasound-guided intercostal nerve cryoablation. Anesthes Analg.

[10] Kim BH, No MY, Han SJ, Park CH, Kim JH (2015). Paraplegia following intercostal nerve neurolysis with alcohol and thoracic epidural injection in lung cancer patient. Korean J Pain.

[11] Jang SH, Ahn SH, Park SM, Kim SH, Lee KH, Lee ZI (2004). Alcohol neurolysis of tibial nerve motor branches to the gastrocnemius muscle to treat ankle spasticity in patients with hemiplegic stroke. Arch Phys Med Rehabil.

[12] Parris D, Fischbein N, Mackey S, Carroll I (2010). A novel CT-guided transpsoas approach to diagnostic genitofemoral nerve block and ablation. Pain Med.

[13] Koyyalagunta D, Engle MP, Yu J, Feng L, Novy DM (2016). The Effectiveness of Alcohol Versus Phenol Based Splanchnic Nerve Neurolysis for the Treatment of Intra-Abdominal Cancer Pain. Pain Physician.

[14] Hung JC, Azam N, Puttanniah V, Malhotra V, Gulati A (2014). Neurolytic transversus abdominal plane block with alcohol for long-term malignancy related pain control. Pain Physician.

[15] Kocabas H, Salli A, Demir AH, Ozerbil OM (2010). Comparison of phenol and alcohol neurolysis of tibial nerve motor branches to the gastrocnemius muscle for treatment of spastic foot after stroke: a randomized controlled pilot study. Eur J Phys Rehabil Med.

[16] Zhao WX, Wang Q, He MW, Yang LQ, Wu BS, Ni JX (2015). Radiofrequency thermocoagulation combined with pulsed radiofrequency helps relieve postoperative complications of trigeminal neuralgia. Genet Mol Res.

[17] Jackson TP, Gaeta R (2008). Neurolytic blocks revisited. Curr Pain Headache Rep.

[18] Lee DG, Jang SH (2012). Ultrasound guided alcohol neurolysis of musculocutaneous nerve to relieve elbow spasticity in hemiparetic stroke patients. NeuroRehabilitation.

[19] Fujita Y (1993). CT-guided neurolytic splanchnic nerve block with alcohol. Pain.

[20] Chen CK, Phui VE, Saman MA (2012). Alcohol neurolysis of lateral femoral cutaneous nerve for recurrent meralgia paresthetica. Agri.

[21] Akural E, Ojala RO, Järvimäki V, Kariniemi J, Tervonen OA, Blanco Sequeiros R (2013). MR-guided neurolytic celiac plexus ablation: an evaluation of effect and injection spread pattern in cancer patients with celiac tumor infiltration. Cardiovasc Intervent Radiol.

[22] Huang W, Li CX, Yan XM, Fang H (2009). UItrastructual changes of neurolytic celiac plexus blocked with different concentration alcohol in rabbit. Pain Clinic Journal.

[23] Jiang CL, Zhang LH, SU F, Qu XL, Luo HB (2008). To observe histological changes of neurolytic celiac plexus block by different concentration of alcohol through posterior-lateral approach in rats in vivo (Chinese). Chinese Journal of Pain Medicine.

[24] Wang BB, Wang BL, Cao ZY, Wang Y (2011). Effects of perineurial block of sciatic nerve with different concentrations of alcohol on structure and function of the nerve and its innervated muscles in rats (Chinese). Chinese Journal of Physical Medicine and Rehabilitation.

